# Prevalence and associated factors of anemia among adolescent girls in Ethiopia: A systematic review and meta-analysis

**DOI:** 10.1371/journal.pone.0264063

**Published:** 2022-03-24

**Authors:** Samuel Derbie Habtegiorgis, Pammla Petrucka, Animut Takele Telayneh, Daniel Shitu Getahun, Lemma Getacher, Simegn Alemu, Molla Yigzaw Birhanu

**Affiliations:** 1 Department of Public Health, College of health Sciences, Debre Markos University, Debre Markos, Ethiopia; 2 College of Nursing, University of Saskatchewan, Saskatoon, Canada; 3 School of Life Sciences and Bioengineering, Nelson Mandela African Institute of Science and Technology, Arusha, Tanzania; 4 Department of Public Health College of Health Science, Debre Berhan University, Debre Birhan, Ethiopia; Georgetown University, UNITED STATES

## Abstract

**Background:**

Anemia is the reduction of red blood cells in size and numbers and an indicator of both poor nutrition and poor health. It is a major global public health problem. Anemia in adolescents and young adults can have negative effects on their cognitive performance and growth. In Ethiopia, previous studies yielded variable prevalence. This review aimed to determine the pooled prevalence of adolescent girls’ anemia and associated factors in Ethiopia.

**Methods:**

We searched for studies reporting anemia and associated factors among adolescent girls as reported in peer reviews publications in Ethiopia from 1988 to 2021 from PubMed, Google Scholar, Web of Science, Science Direct, Cochrane Library, and Worldwide Science database. The search strategy identified 309 cross-sectional studies. After screening for potentially eligible articles, we identified 37 publications for full text review, following which 10 publications were included in the final review. Using data from the review, we performed meta-analysis to produce pooled estimates and assess the prevalence of anemia and associated risk factors. Data were extracted using a standardized data extraction format prepared in Microsoft Excel™ and transferred to Stata ™ Version 14.0 for management and further analysis. To identify the source of heterogeneity, subgroup analysis using sample size and study setup was computed, and I^2^ test was used to declare the presence or absence of significant heterogeneity during subgroup analysis. A random-effect meta-analysis model was used to estimate the pooled prevalence of adolescent girls’ anemia. Moreover associated factors for adolescent anemia were assessed too.

**Results:**

The overall pooled prevalence of anemia among adolescent girls’ in Ethiopia was 23.02% (95% CI: 17.21to 28.84). In the subgroup analysis, studies that have a higher sample size than mean have a higher pooled prevalence (27.35%) (95% CI: 21.42 to 33.28) compared to their counterparts. Age being 15–19 (OR: 2.13; 95% CI: 1.52 to 2.96), living in rural areas (OR: 2.05; 95% CI: 1.66 to 2.54), and low dietary diversity (OR: 1.35; 95% CI: 1.00 to 2.34), were the identified factors associated with anemia among adolescent girls’.

**Conclusion:**

The pooled prevalence of anemia among adolescent girls in Ethiopia was moderately high. Being in 15–19 years, rural residence, and low dietary diversity score were found to be the significant factors of anemia among adolescent girls in Ethiopia.

## Background

Anemia is a medical complication in which the number and size of red blood cells, or the hemoglobin concentration, falls below the reference range. It has the potential consequence of impairing or reducing the capacity of the blood to transport oxygen throughout the body [1–3]. Anemia is resulting from both poor nutritional status and/or poor health condition.

During adolescence, anemia is mostly caused by frequent nutritional problem due to their rapid growth and physical changes, high iron requirements, high rate of infection and worm infestation, as well as the early marriage and adolescent pregnancy [1, 2, 4].

Anemia in adolescents can have negative effects on their cognitive performance and growth and pregnancy during adolescence, can increase maternal morbidity and mortality as well as poor birth outcomes [2]. Furthermore, through its effect on cognitive and work performance, anemia can impact current and future economic productivity of the country at large [2, 5]. Initiatives to prevent anemia commonly targets to infants, young children, pregnant, and lactating women but not focusing on adolescents due to this the consequences of anemia among adolescents remains as a big deal [3, 6].

Globally the most significant cause of anaemia is iron-deficiency (ID). The onset of anemia secondary to iron deficiency is generally assumed to account 50% of anemia occurred in the world [7].

Heavy blood loss, acute and chronic infections, low intake of iron, poor absorption of iron, parasite infections (malaria, tuberculosis, and Human Immunodeficiency Virus (HIV)) can lower blood hemoglobin concentrations as well as vitamin A and B_12_, folate, riboflavin, and copper deficiencies can also increase the risks of anemia [7, 8].

Different studies have shown the prevalence of adolescent girls’ anaemia ranges from 78.5 to 78.8% [9, 10]. To determine the prevalence and associated factors of anemia among adolescent girls in Ethiopia was conducted previously and it is ranging from 8.7% [11] to 39.0% [12]. This study aimed to estimate the pooled prevalence and factors associated with anemia among adolescent girls in Ethiopia due to the presence of heterogeneity in the prevalence and inconsistency of associated factors. Finally this study concludes by discussing the implication of these findings for health public policy on management of anemia among adolescent girls in Ethiopia. Additionally, it will have importance for clinicians and future researchers in related topics.

## Methods

### Exploration approach of primary studies

We searched articles on the topic from Web of Science, Science Direct, PubMed, Cochrane library, google Scholar and Worldwide Science databases. The keywords on the condition, context and population were combined using Boolean words for searching: As result, “(Adolescents) OR (Adolescence) OR (Teens) OR (Teen) OR (Teenagers) OR (Teenager) OR (Youth) OR (Youths) OR (Adolescents) OR (Female Adolescent) OR (Female Adolescent)) OR (female adolescents)) OR (Adolescents[MeSH Terms])) OR (Adolescence[MeSH Terms]) OR (Teens[MeSH Terms]) OR (Teen[MeSH Terms]) OR (Teenagers[MeSH Terms]) OR (Teenager[MeSH Terms]) OR (Youth[MeSH Terms]) OR (Youths[MeSH Terms]) OR (Adolescents[MeSH Terms]) OR (Female Adolescent[MeSH Terms]) OR (female adolescents[MeSH Terms]) AND ((Ethiopia) OR (Federal republic of Ethiopia) OR (ethiopia[MeSH Terms]) AND (Anemia) OR (Anaemia) OR (Aenemia)) OR (anaemia)) OR (Anemia[MeSH Terms]) OR (Iron-Deficiency[MeSH Terms])”. After the two authors (SDH &DS) were independently searching the primary articles, the presence of conflicts regarding the included articles was solving by discussing and reaching on consensus in the presence of third author (MYB). MYB and SDH extracted the data from the included articles using standard Microsoft Excels and exported into Stata version 14.0 for management and further analysis. This systematic review and Meta-analysis was presented according to the Preferred Reporting Items for Systematic Reviews and Meta-Analyses (PRISMA) [13].

### Eligibility criteria

#### Inclusion criteria

The observational studies conducted on the prevalence and associated factors of anemia among adolescent girls in English which is published in English were included.

#### Exclusion criteria

After frequent request of the cross ponding authors, articles with incomplete information or were excluded. Reports and reviews were also excluded from this study.

#### Data extraction

We extracted all the necessary data using a data extraction format prepared in Microsoft Excel independently. For the primary outcome, the data extraction format included first author, region (according to Ethiopian political administration), area-specific place where the study took place, study period, publication year, study design, sample size, response rate, and prevalence of adolescent girls’ anemia.

For the second outcome (associated factors), the data extraction format was prepared for each specific factor (age, residence, dietary diversity score, source of water, household food security, living, and family size). For each factor, we extracted the data in the form of two by two tables to calculate the odds ratio. Any disagreements between the two authors during extraction were solved by consensus.

#### Outcome measurements

This study has two outcomes: the primary outcome was to determine the prevalence, while the secondary outcome was to identify the factors associated with anemia among adolescent girls. Concerning factors variables, we calculated the odds ratio from the primary studies using the two by two tables.

#### Quality assessment

We used the Newcastle-Ottawa Scale tool to assess the quality of the included cross-sectional studies [14]. The tool consisting of three main sections: 1) assesses the methodological quality of the study; 2) evaluates the comparability of the studies; and 3) measures the outcome measurement and statistical analysis quality of the original articles.

#### Statistical analysis

The data were extracted using a Microsoft Excel™ format, and the data were exported from Microsoft Excel to Stata™ Version 14.0 (software) for management and further analysis. The data were presenting using a text, table, graph and forest plot. We calculated the standard error of prevalence for each original article.

The heterogeneity between the prevalence of the previous studies was checked by using the I^2^ test together with the p-values. Due to the presence of significant heterogeneity between previous studies (I^2^ = 96.5%, p <0.001), a random-effects Meta regression analysis model was used to estimate (Der Simonian and Laird’s) pooled prevalence of anemia among adolescent in Ethiopia. Moreover, the univariate meta-regression model considered the publication year and the sample size of the studies to identify the possible source of heterogeneity. However, none of them was statistically significant. Potential publication bias was also assessed by using Egger’s correlation and Begg’s regression intercept tests at a 5% significant level respectively. The outcomes of these tests revealed that there is no significant publication bias (p>0.664 in Egger’s test). To minimize the heterogeneity of the original study, subgroup analysis was done based on the mean sample size and study setup.

## Results

### Exploration of original articles

**Fig 1:**
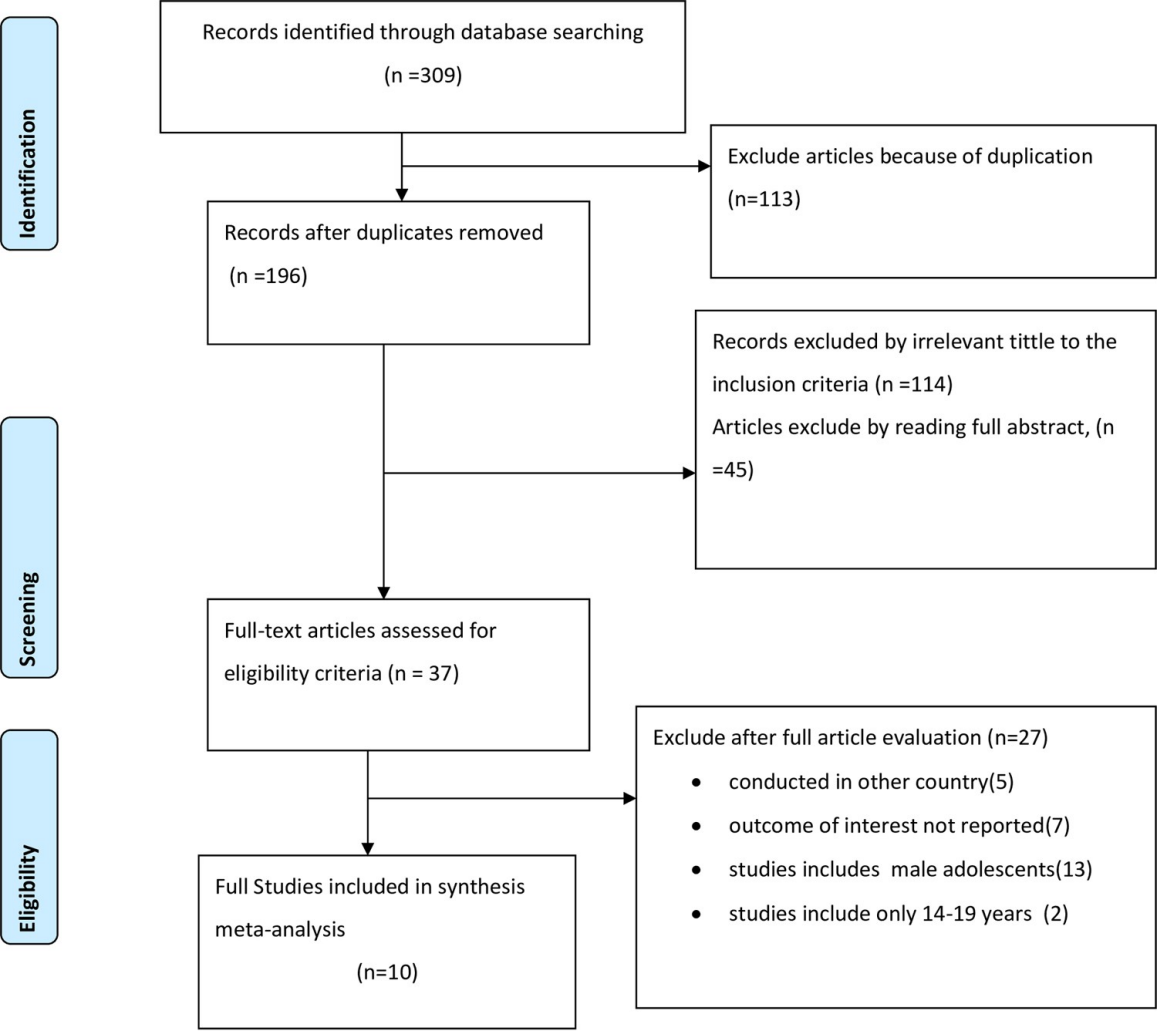


From a total of 309 articles identified using the above-mentioned search database engines, 113 articles were excluded due to duplication. By reading the title and abstracts 159 articles were excluded from the remaining 196 articles as irrelevant to this review. By assessing the 37 full texts of the article, only 10 articles fulfilled the inclusion criteria and are included in this Systematic review and Meta-analysis (**Fig 1**).

### Characteristics of original articles

In this study, 10 original articles met the inclusion criteria and were included. All of the articles included in this review were cross-sectional studies, and the sample size of the studies ranged from 257 in Huruta district [11] to 1323 in Debrelibanos, Damotegale, and Laygaynt districts [15]. The pooled prevalence of anemia among adolescent girls in Ethiopia was estimated using 5,547 participants. The included articles were done in five regions of Ethiopia. Among those, one from Addis Ababa [16], four from Oromia [11, 12, 17, 18], two from Amhara [19, 20], one from the Somali region [21], one from SNNPR [22], and the remaining one was done at Debrelibanos, Damotegale, and Laygaynt districts [15]. The highest and the lowest prevalence of anemia among adolescent girls were reported from Ambo, Oromia region (39%) [12], and Huruta district Oromia (8.7%) [11] respectively.

The response rates in the included primary studies was ranged from 91% in Bible, Oromia [18] to 100% in Demba, Amhara [19], and Huruta district, Oromia [11]. Among the included articles, nine articles were published and presented in the major databases and one article was found online in the form of research square (**Table 1**).

**Table 1:** Characteristics of 10 studies reporting the prevalence of anemia among adolescent girls in Ethiopia, 2021.

Authors	study period	Pub/year	Area	Region	Sample	Response rate %	Prevalence (95% CI)
Regasa *et al* [17]	Feb-Mar	2019	WayuTuqa	Oromia	454	98	27(22.92,31.08)
Teji *et al* [18]	Dec-Feb	2016	Babile	Oromia	600	91	32(28.27,35.73)
Seyoum *et al* [11]	NA	2019	Huruta	Oromia	257	100	8.7(5.25,12.15)
Gonete *et al* [19]	Mar1-Apr	2018	Dembia	Amhara	462	100	25.5(21.53,29.47)
Mengistu *et al* [20]	Mar -Apr	2019	Bahir Dar	Amhara	443	95.5	11.1(8.17,14.03)
Engidaw *et al* [21]	Mar-Apr	2018	Aw-bere	Somali	456	95.83	22(18.20,25.80)
Demelash *et al* [16]	Mar-Apr	2019	AA	AA	594	95.9	21.1(17.82,24.38)
Tura, MR et al [12]	August	2020	Ambo	Oromia	551	95	39(34.93,43.07)
Alemu T, et al [22]	January	2020	Hadero	SPNN	407	98.2	15.2(11.71, 18.69)
Gebreyesus *et al* [15]	Oct-Dec	2019	*	Ethiopia	1323	99.85	29(26.55,31.45)

* = Debrelibanos, Damotegale and Laygaynt districts.

During the quality assessment of the included articles, the quality score was ranging from 5–8 out of 10 points.

### Data management and analysis

**Fig 2:**
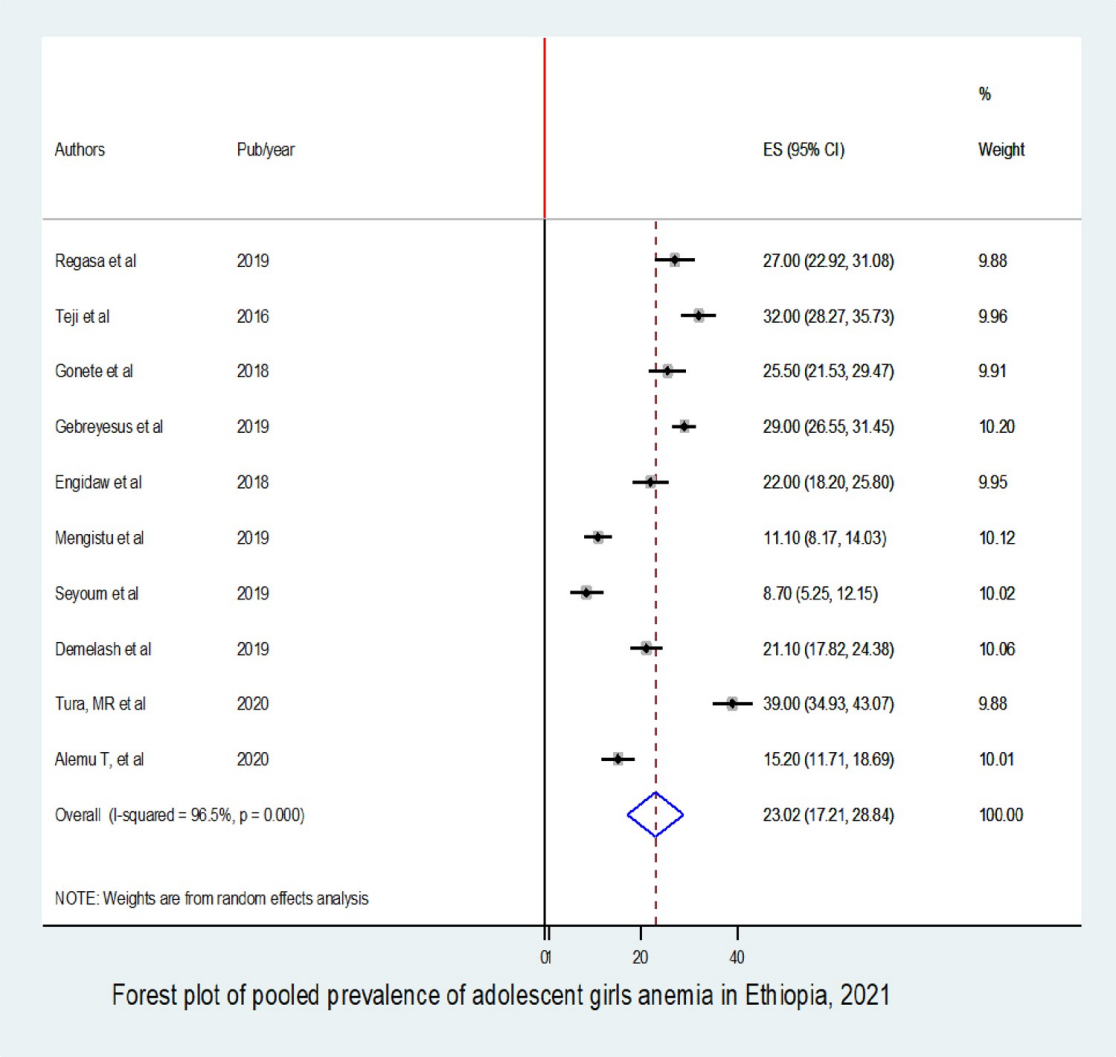


The overall pooled prevalence of anemia in adolescent girls in Ethiopia was 23.02% (95% CI: 17.21, 28.84) (**Fig 2**). Due to the presence of statistical significant heterogeneity between studies (I^2^ = 96.5%, p≤0.001), we employed a random effect meta-analysis model, to estimate the pooled prevalence of anemia among adolescent girls in Ethiopia. To detect the possible sources of heterogeneity, we were investigated possible factors associated with the heterogeneity such as sample size variation and publication year by using meta-regression models, but none were found to be statistically significant (**Table 2**).

**Table 2:** Factor, related to the heterogeneity of adolescent girls’ anemia in the current meta-analysis (univariate meta-regression model).

Variables	Coefficient	p-value
Sample size	0.015	0.19
Publication year	-1.683	0.57

**Fig 3:**
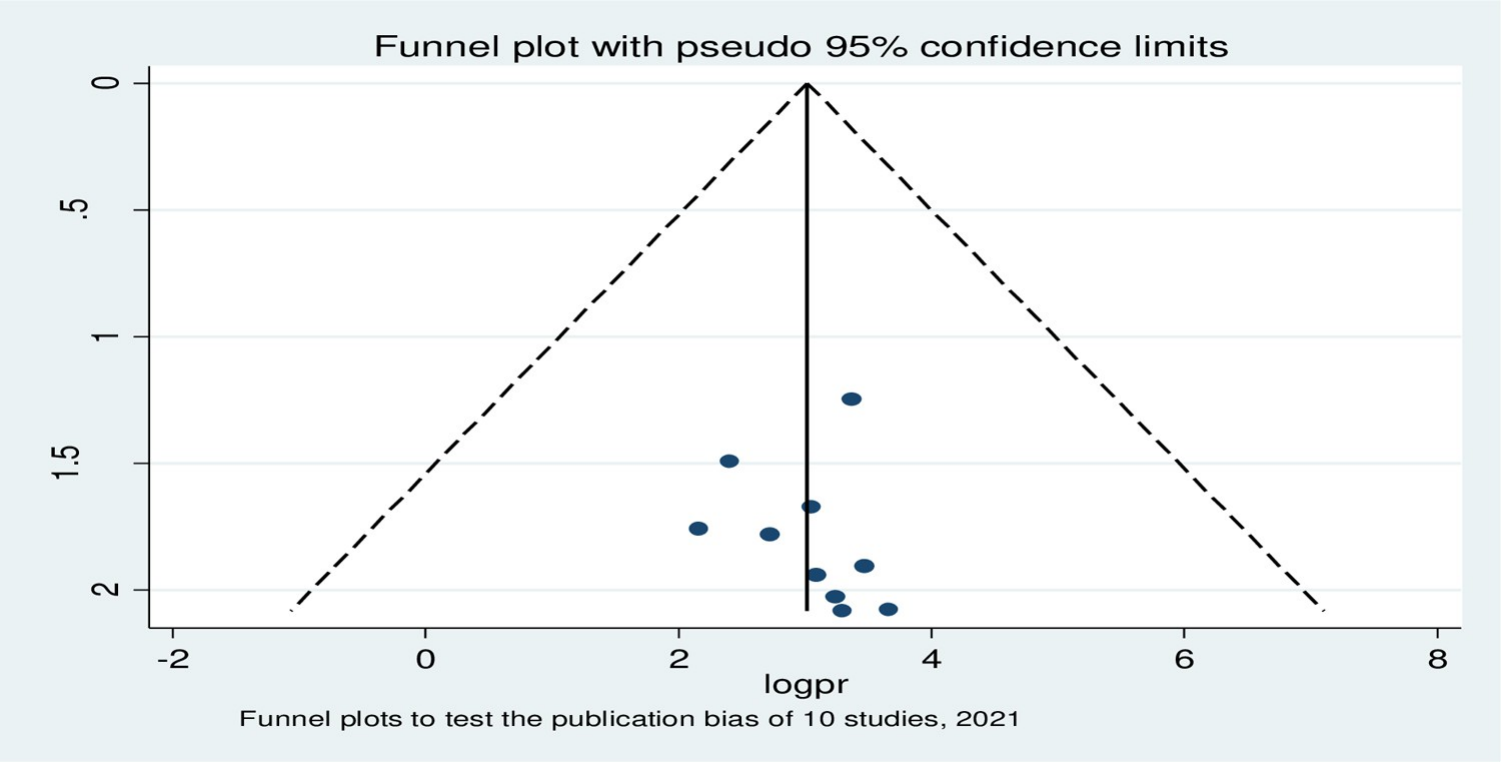


To assess publication bias, funnel plot was used and it was asymmetry by visual inspection and it appeared quite asymmetrical (**Fig 3**) indicating the presence of publication bias. Then after Egger’s and Begg’s test were computed to show whether this publication bias was significant or not. As a result, the eggers and Begg’s test value were (p > 0.664, p>0.210,) respectively. Therefore, the observed publication bias was not significant, it was due to chance.

### Subgroup analysis

We performed subgroup analysis based on the mean sample and study setup. The subgroup analysis showed that studies that have a higher sample size than the sample mean showed higher prevalence 27.35% (95%CI: 21.42–33.28) (**Table 3**).

**Table 3:** Subgroup prevalence of adolescent girls anemia in Ethiopian, 2020 (n = 10).

Variables	Characteristics	No. of studies	No. of participants	Prevalence
mean sample size	Above	3	2517	27.35(21.42–33.28)
	Below	7	3030	21.16(13.50,28.82)
Study setup	Institution	3	1510	24.36(20.72, 28.01)
	Community	7	4037	22.40(14.28,30.52)

Furthermore, the prevalence of anemia among adolescent was found to be slightly higher than among institution-based studies with a pooled prevalence of 24.36% (20.72, 28.01) (**Table 3**).

### Associated factors to adolescent girls anemia in Ethiopia

To identify associated factors of anemia among adolescent girls’ seven studies [12, 15, 17–19, 21, 22] were included. From the ten primary studies three predictive factors were identified.

### Age category of study participants

**Fig 4:**
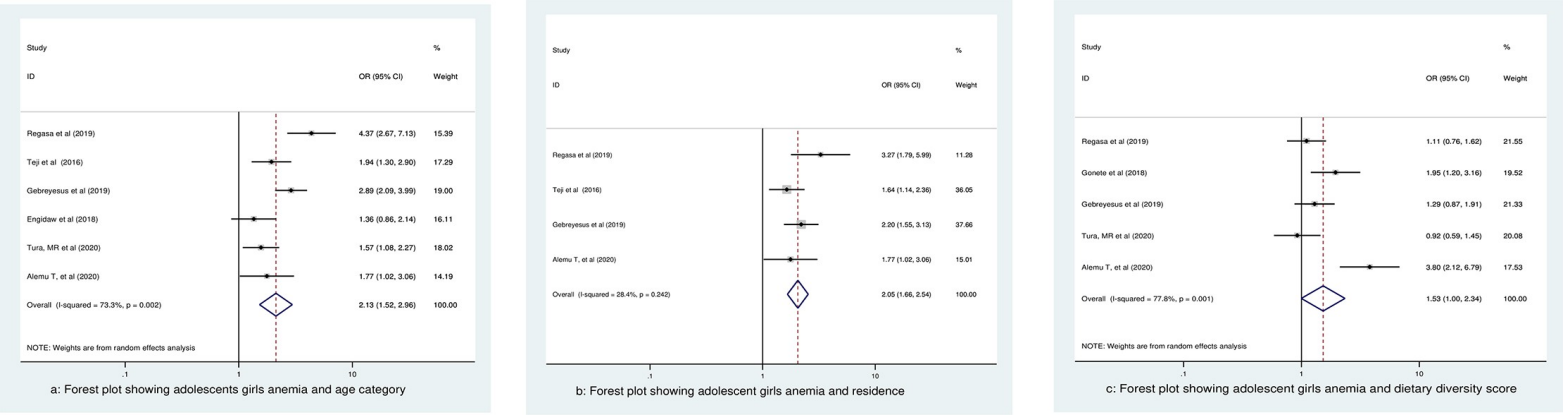


To determine the association between the age with anemia among adolescent girls six articles [12, 15, 17, 18, 21, 22] with 3,791 study participants were included. Hence, there was a significant association between age and anemia adolescent girls. Those girls who were 15 to 19 years old were about 2.13 times more likely to be anemic (OR: 2.13; 95% CI: 1.52 to 2.96) as compared to those between 10 to 14 years old (**Fig 4A**).

### Residence and anemia among study participants

To determine the association of residence and anemia, four articles with 2,784 study participants were included [15, 17, 18, 22]. The analysis showed a significant association with rural dwellers were more likely to develop anemia (OR: 2.05; 95% CI: 1.66to 2.54) as compared urban dwellers (**Fig 4B**).

### Dietary diversity score and anemia

In the analysis of the association of dietary diversity score and anemia, we considered five articles with a total of 3,197 study participants [12, 15, 17, 19, 22]. Households with low dietary diversity score were more likely to exhibit anemia 47% (OR: 1.35; 95%CI: 1.00, 2.34) as compared to households with adequate dietary diversity (**Fig 4C**).

## Discussion

Generally, the pooled prevalence of anemia among adolescent girls in Ethiopia was 23.02% (95% CI: 17.21, 28.84). This finding of anemia among adolescent girls was lower than the studies conducted in India and Far East countries [9, 23–26], in Eastern Sudan [27]. However, this finding was higher than many studies conducted in Turkey (8.3%), in Denizli State Hospital [28], in Kavar Urban Area, Southern Iran (5.8%) [29], and in China (7.4%) [30]. Discrepancies may be a consequence of variations in study participants, socio demographic characteristics, sample size, study period, and time.

This study also identified factors associated with anemia among adolescent girls using the report of primary studies. In this review, being in 15–19 years, living in rural and low dietary diversity score were the identified factors associated with anemia among adolescent girls in Ethiopia.

Adolescents’ girls aged in 15–19 years were two times more likely to be anemic as compared as 10 to 14 years. This finding is supported by studies conducted in Bhutan, South Asia [31] in Siaya District, Kenya [32], Wayu Tuqa district, southwest Ethiopia [17]. This might be due to the onset of menstruation, greater likelihood of pregnancy and abortion for those 15–19 years as compared to 10 to 14 years. In addition to the above, there might be the fast physical change and development in this age group may be the possible reason. The odds of anemia occurrence among rural adolescent girls was 2.05 times more likely as compared to urban residents. It is supported by demographic health survey in adolescent girls 2000–2017 [1]. Conversely, this finding was contradicted in studies conducted in Nepal [23], and Bhutan [31]. The discrepancy of these results might be related to a lack of awareness in a rural area of Ethiopia since most adolescent girls of the country are rural residents. Furthermore, this might be due to the low diet diversity the in rural resident of the country (Ethiopia), since anemia might be resulted secondary to poor dietary diversity.

Finally, household dietary diversity score was found to be a significant factor associated with anemia adolescent girls. Those households with low dietary diversity scores were 47% times more likely to experience anemia among adolescent girls as compared to those households who had adequate dietary diversity. This finding was consistent with studies conducted in Latin America and Caribbean countries and Kenya [32, 33]. This might be due to the fact that increasing food variety and anemia, especially in this age group, adolescent girls start menstruation which leads to anemia due to loss of blood. Adolescent girls who lack access to an adequate variety of foods become anemic because of low content of iron, vitamin B_12_, or folate that is used for hematopoiesis especially Erythropoiesis.

### Limitations of the study

As the drawback of this study, all the included studies were taken from five region of the country Ethiopia, which might not be represent the actual figure of anemia among adolescent girls. As well as all studies included in this review were cross-sectional in design so may lack representativeness of the seasonal variation of anemia status among adolescent girls, and not to establish a causal temporal relationship due to the snapshot nature of the design. In addition to the above, only articles published in English language were included in this review.

## Conclusion

In this review, the pooled prevalence of anemia among adolescent girls in Ethiopia was moderately high. Age, residence, and dietary diversity score were identified as statistically significant factors of anemia among adolescent girls in Ethiopia.

It is strongly recommended that those adolescent girls aged 15–19 and rural resident should get iron supplementation to prevent their experience of anemia. As well as health education to promote health especially on dietary diversity should be given for adolescent girls to overcome the existence of anemia.

## Supporting information

S1 Table Key words and Mesh terms used for searching primary studies of prevalence of anemia and associated factors among adolescent girls in Ethiopia. (DOCX)

S2 Table Details of primary studies searching history for meta-analysis and review. (DOCX)

S3 Table PRISMA 2009 checklist preferred reporting items for systemic reviews. (DOCX)

## List of abbreviations

HB: haemoglobin
HIV: human immune virus
IDA: iron deficiency anemia
WHO: World Health Organization
