# Supplementary material for: Prevalence and associated factors of anemia among adolescent girls in Ethiopia: A systematic review and meta-analysis
Source: PLoS One. 2022 Mar 24;17(3):e0264063. doi: 10.1371/journal.pone.0264063 (PMC8947116; doi:10.1371/journal.pone.0264063)
Supplement: S1 Table — (DOCX) [file pone.0264063.s001.docx]

| Condition | Context | Population |
| --- | --- | --- |
| Key words  Anemia  Anaemia  Hemoglobin | Key words  Ethiopia  Federal republic of Ethiopia | Keywords  Adolescents  Adolescence  Teens  Teen  Teenagers  Teenager  Youth  Youths  Adolescents, Female  Adolescent, Female  Female Adolescent  Female Adolescents |
| MeSH  Anemia  Iron-Deficiency | MeSH  ethiopia | MeSH  Adolescents  Adolescence  Teens  Teen  Teenagers  Teenager  Youth  Youths  Adolescents, Female  Adolescent, female  Female Adolescent  Female Adolescents |

**S1**

Logic grid of CoCoPo
