# Supplementary material for: Prevalence and associated factors of anemia among adolescent girls in Ethiopia: A systematic review and meta-analysis
Source: PLoS One. 2022 Mar 24;17(3):e0264063. doi: 10.1371/journal.pone.0264063 (PMC8947116; doi:10.1371/journal.pone.0264063)
Supplement: S2 Table — (DOCX) [file pone.0264063.s002.docx]

**S2**

## History and Search Details

Top of Form

Download

Bottom of Form

Delete

| **Search** | **Actions** | **Details** | **Query** | **Results** | **Time** |
| --- | --- | --- | --- | --- | --- |
| #4 |  |  | Search: **(((((((((((((((((((((((((Adolescents) OR (Adolescence)) OR (Teens)) OR (Teen)) OR (Teenagers)) OR (Teenager)) OR (Youth)) OR (Youths)) OR (Adolescents)) OR (Female Adolescent)) OR (Female Adolescent)) OR (Female Adolescents)) OR (Adolescents[MeSH Terms])) OR (Adolescence[MeSH Terms])) OR (Teens[MeSH Terms])) OR (Teen[MeSH Terms])) OR (Teenagers[MeSH Terms])) OR (Teenager[MeSH Terms])) OR (Youth[MeSH Terms])) OR (Youths[MeSH Terms])) OR (Adolescents[MeSH Terms])) OR (Female Adolescence[MeSH Terms])) OR (Female Adolescent[MeSH Terms])) OR (female adolescents[MeSH Terms])) AND (((Ethiopia) OR (Federal republic of Ethiopia)) OR (ethiopia[MeSH Terms]))) AND (((((Anemia) OR (Anaemia)) OR (Hemoglobin)) OR (Anemia[MeSH Terms])) OR (Iron-Deficiency[MeSH Terms]))** | [242](https://pubmed.ncbi.nlm.nih.gov/?term=%28%28%28%28%28%28%28%28%28%28%28%28%28%28%28%28%28%28%28%28%28%28%28%28%28Adolescents%29+OR+%28Adolescence%29%29+OR+%28Teens%29%29+OR+%28Teen%29%29+OR+%28Teenagers%29%29+OR+%28Teenager%29%29+OR+%28Youth%29%29+OR+%28Youths%29%29+OR+%28Adolescents%29%29+OR+%28Female+Adolescent%29%29+OR+%28Female+Adolescent%29%29+OR+%28Female+Adolescents%29%29+OR+%28Adolescents%5BMeSH+Terms%5D%29%29+OR+%28Adolescence%5BMeSH+Terms%5D%29%29+OR+%28Teens%5BMeSH+Terms%5D%29%29+OR+%28Teen%5BMeSH+Terms%5D%29%29+OR+%28Teenagers%5BMeSH+Terms%5D%29%29+OR+%28Teenager%5BMeSH+Terms%5D%29%29+OR+%28Youth%5BMeSH+Terms%5D%29%29+OR+%28Youths%5BMeSH+Terms%5D%29%29+OR+%28Adolescents%5BMeSH+Terms%5D%29%29+OR+%28Female+Adolescence%5BMeSH+Terms%5D%29%29+OR+%28Female+Adolescent%5BMeSH+Terms%5D%29%29+OR+%28female+adolescents%5BMeSH+Terms%5D%29%29+AND+%28%28%28Ethiopia%29+OR+%28Federal+republic+of+Ethiopia%29%29+OR+%28ethiopia%5BMeSH+Terms%5D%29%29%29+AND+%28%28%28%28%28Anemia%29+OR+%28Anaemia%29%29+OR+%28Hemoglobin%29%29+OR+%28Anemia%5BMeSH+Terms%5D%29%29+OR+%28Iron-Deficiency%5BMeSH+Terms%5D%29%29&sort=) | 04:07:15 |
